# The Role of the Novel Exopolyphosphatase MT0516 in *Mycobacterium tuberculosis* Drug Tolerance and Persistence

**DOI:** 10.1371/journal.pone.0028076

**Published:** 2011-11-21

**Authors:** Seema M. Thayil, Norman Morrison, Norman Schechter, Harvey Rubin, Petros C. Karakousis

**Affiliations:** 1 Department of Medicine, Johns Hopkins University School of Medicine, Baltimore, Maryland, United States of America; 2 Department of Medicine, University of Pennsylvania School of Medicine, Philadelphia, Pennsylvania, United States of America; 3 Department of International Health, Johns Hopkins Bloomberg School of Public Health, Baltimore, Maryland, United States of America; French National Centre for Scientific Research - Université de Toulouse, France

## Abstract

Inorganic polyphosphate (poly P) has been postulated to play a regulatory role in the transition to bacterial persistence. In bacteria, poly P balance in the cell is maintained by the hydrolysis activity of the exopolyphosphatase PPX. However, the *Mycobacterium tuberculosis* PPX has not been characterized previously. Here we show that recombinant MT0516 hydrolyzes poly P, and an MT0516-deficient *M. tuberculosis* mutant exhibits elevated intracellular levels of poly P and increased expression of the genes *mprB*, *sigE*, and *rel* relative to the isogenic wild-type strain, indicating poly P-mediated signaling. Deficiency of MT0516 resulted in decelerated growth during logarithmic-phase in axenic cultures, and tolerance to the cell wall-active drug isoniazid. The MT0516-deficient mutant showed a significant survival defect in activated human macrophages and reduced persistence in the lungs of guinea pigs. We conclude that exopolyphosphatase is required for long-term survival of *M. tuberculosis* in necrotic lung lesions.

## Introduction

It is estimated that 2 billion people worldwide have latent tuberculosis (TB) infection, representing a vast potential reservoir for subsequent reactivation disease, particularly in the setting of the HIV pandemic [1]. Latent TB infection is believed to result from the immunological control of a small number of nonreplicating and slowly metabolizing organisms [2], which have adapted to the unfavorable microenvironmental conditions within lung granulomas [3], likely including hypoxia [4], nutrient limitation, and acidic pH [5]. These “dormant” bacilli exhibit phenotypic tolerance to bactericidal antibiotics such as isoniazid [6], which inhibits the mycolic acid synthesis pathway required for cell wall synthesis [7], [8]. Although previous studies have highlighted the importance of various adaptive mechanisms in promoting the long-term persistence of *M. tuberculosis* (Mtb) in host tissues, including the stringent response [9], [10] and a switch to utilization of fatty acids as a source of carbon and energy through the glyoxylate cycle [11], the Mtb molecular pathways underlying latency remain largely undefined.

Inorganic polyphosphate (poly P), a linear polymer of many tens or hundreds of inorganic phosphate residues linked by high-energy phosphoanhydride bonds, has been postulated to play a regulatory role in the transition to bacterial dormancy [12]. In bacteria, poly P is synthesized by polyphosphate kinase (PPK), which catalyzes the reversible transfer of the terminal (γ) phosphate of ATP to poly P [13]. The exopolyphosphatase PPX is involved in poly P degradation and contributes to the maintenance of the poly P dynamic balance in the cell [14]. During the *Escherichia coli* stringent response, intracellular poly P levels increase significantly as a result of inhibition of PPX activity by the alarmone (p)ppGpp [14], which is synthesized by Rel when uncharged tRNA binds nonenzymatically to the acceptor site of an elongating ribosome stalled at a codon on an mRNA, signaling that the environment is limited in amino acids [15]. Poly P induces expression of the *rpoS* gene [16], [17], which is also regulated by (p)ppGpp, and the encoded RNA polymerase sigma factor RpoS directs the transcription of >50 genes involved in cascades downshifting growth and metabolism, thereby adjusting the cell to a persistent state [18].

Recently, poly P has been shown to play an important role in mycobacterial survival under various growth-limiting conditions. A *ppk1*-deficient mutant of *M. smegmatis* was found to be defective in poly P synthesis and more sensitive to surface stress, oxidative stress, and extended anaerobic incubation, and a Rv2984 (*ppk1*)-knockdown strain of Mtb with reduced intrabacillary poly P content exhibited defective survival in THP-1 macrophages [19]. During stationary phase, *ppk1* expression increases in *M. smegmatis* [19], suggesting that poly P accumulation in mycobacteria may be at least partially attributable to an increase in its biosynthetic rate. In mycobacteria, poly P appears to induce the *mprAB-sigE-rel* signaling pathway [19], [20], indicating that poly P can control (p)ppGpp synthesis through transcriptional regulation of *rel*.

We reasoned that Mtb must also have a PPX capable of poly P hydrolysis, as in other bacteria [14], in order to maintain a dynamic balance of intracellular poly P. Bioinformatic searches predicted that the gene Rv0496/MT0516 encodes an enzyme with exopolyphosphatase activity, which was confirmed experimentally in the current study. An MT0516-deficient recombinant mutant (MT0516::Tn) was generated to study the role of PPX in Mtb growth in axenic cultures and in human macrophages, as well as the effect of PPX deficiency on antibiotic tolerance. To determine the impact of poly P accumulation on Mtb pathogenesis in the animal host, we studied the growth, survival, and pathology of the MT0516-deficient mutant in the guinea pig model of chronic TB infection.

## Results

### Homology-based identification of putative M. tuberculosis exopolyphosphatases

**Figure 1:**
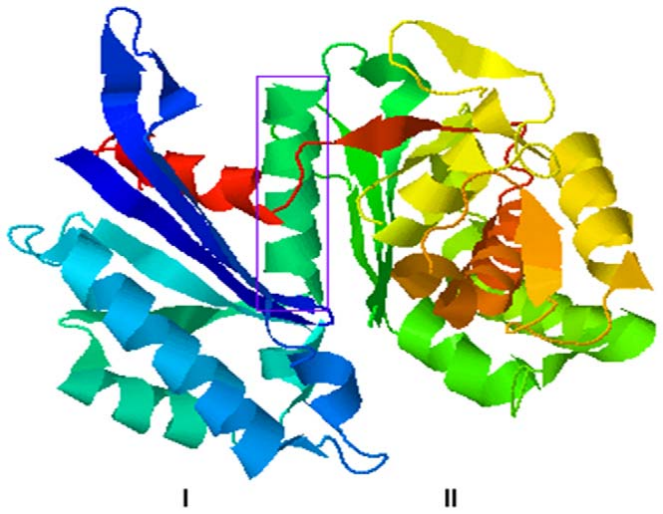
Protein modeling predicts that *M. tuberculosis* MT0516 is an exopolyphosphatase. The protein backbone ribbon structure was modeled by PHYRE [23], showing the conserved hydrolase fold associated with exopolyphosphatases. Left-angled, top-down view of the interface canyon between domains I and II with the α helix 4 (within box) that houses the highly conserved active site E112 side chain opening into the canyon floor. The canyon walls are lined with β sheets.

Based on homology searches of the *M. tuberculosis* genome [21], the genes Rv0496/MT0516 and Rv1026/ MT1054 were predicted to encode exopolyphosphatases, consistent with earlier predictions by Lindner et al. [22]. Pfam HMM E-values (2.2e^−94^ and 1.2e^−91^, respectively) localized the proteins to the 2065-membered single-domain Ppx-GppA family (PF02541), which is global among prokaryotes. Three dimensional structural modeling against the PHYRE template database [23] was used to predict the most likely PPX to hydrolyze poly P. MT0516 has structural homology to the first 2 domains of *E. coli* O157:H7 PDB 2FLO at 100% precision [24] and E-value 3e^−40^ (Fig. 1) while MT1054 has structural homology to *Aquifex aeolicus* PDB 2J4R at 100% precision [25] and E-value 1.2e^−38^. We hypothesized that MT0516, a nonessential gene, encoded an exopolyphosphatase hydrolyzing poly P, whereas MT1054, an essential gene, encoded a (p)ppGppase. It is of interest that both genes are present in the *M. leprae* minimal genome required for slow growth in human tissues [26].

### Deficiency of MT0516 leads to poly P accumulation, growth restriction, and antibiotic tolerance

**Figure 2:**
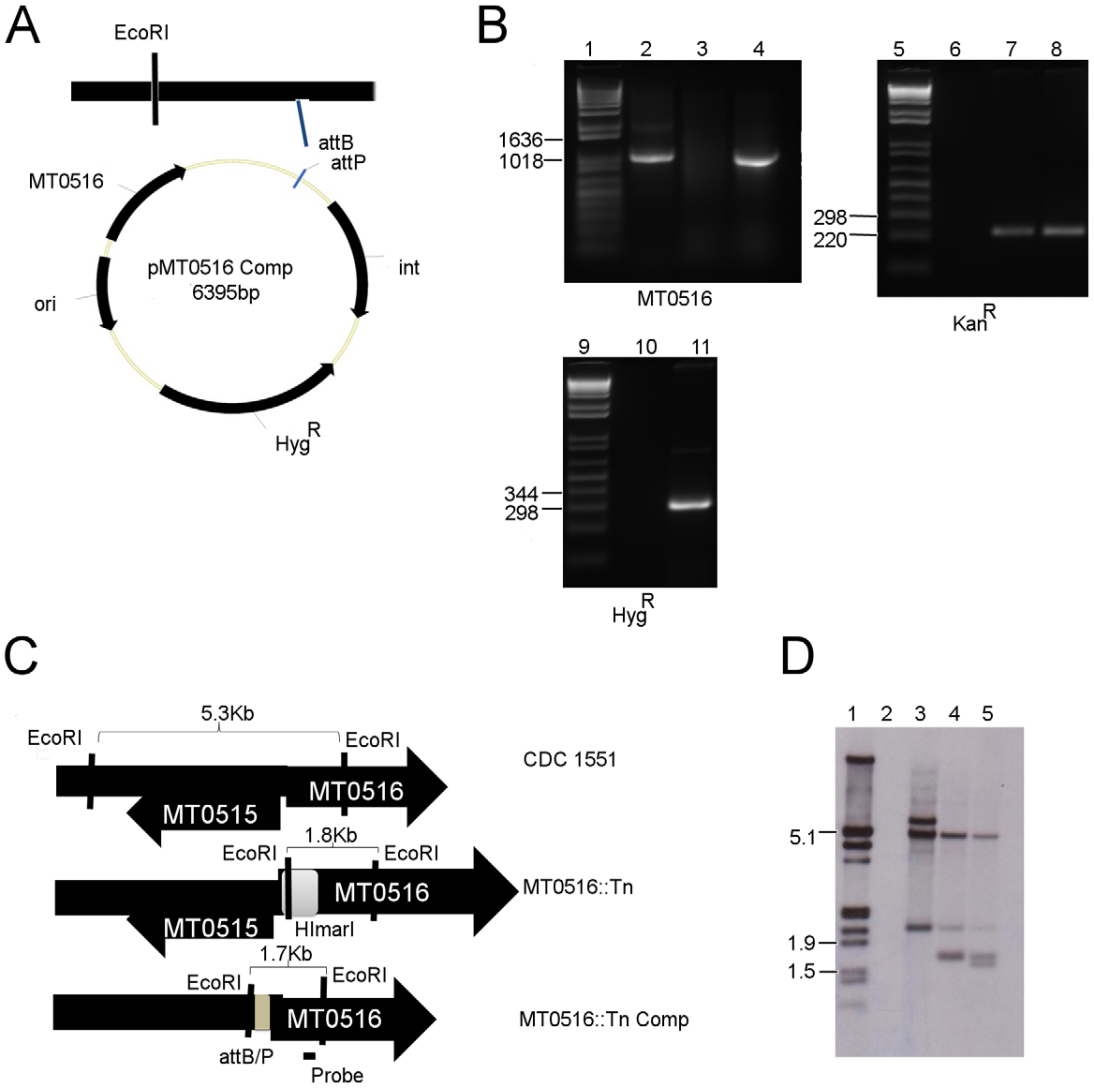
Complementation of transposon mutant MT0516::Tn. A. Diagram of expected recombination between integrating plasmid and genomic DNA. B. PCR analysis of genomic DNA. A list of relevant primers is included in Table 1. Lane 1: Molecular weight markers. Lanes 2-4: Amplification of the MT0516 gene from wild-type, MT0516::Tn, and MT0516::Tn Comp, respectively. The expected 1035-bp PCR product is present in Lanes 2 and 4 but absent in Lane 3. Lane 5: Molecular weight markers. Lanes 6-8: Amplification of the kanamycin resistance cassette from wild type, MT0516::Tn and MT0516::Tn Comp, respectively, yielding the expected 226-bp product only in Lanes 7 and 8. Lane 9: Molecular weight markers. Lanes 10-11: Amplification of the hygromycin resistance cassette from MT0516::Tn and MT0516::Tn Comp, respectively, yielding the expected 319-bp product only in Lane 11. C. Diagram of expected sizes of *EcoRI*-digested genomic fragments expected to hybridize to probe recognizing region of MT0516 coding sequence by Southern blot (D). Note that MT0516::Tn Comp is expected to have two different size fragments hybridizing to the MT0516 probe, including that found in MT0516::Tn (1.8 Kb) and a distinct fragment unique to the attB integration site (1.7 Kb). D. Southern blot detecting the presence of DNA fragments bound to the MT0516 hybridization probe. Lane 1: Molecular weight markers. Lane 2: Blank. Lanes 3-5: Wild type, MT0516::Tn, and MT:0516::Tn Comp, respectively.

In order to test the role of MT0516 in *M. tuberculosis* regulation of poly P content and bacillary growth, an MT0516-deficient mutant (MT0516::Tn) previously generated using transposon mutagenesis of Mtb CDC1551 [27] was used in this study. The MT0516 gene and flanking sequences were then cloned into MT0516::Tn to generate a complement strain (MT0516 Comp; Fig. 2A), which was used to confirm that mutant phenotypes were in fact attributable to MT0516 deficiency. PCR amplification of a region of MT0516 revealed a product of the correct size in wild type and MT0516 Comp strains (Fig. 2B). Additionally, MT0516 Comp can be distinguished genetically from wild type by the presence of a kanamycin-resistance cassette in the former, which is also present in MT0516::Tn, and by the presence of a unique hygromycin-resistance cassette (Fig. 2B). Integration of MT0516 at the proper location is expected to produce a 1.8-kb restriction fragment compared to a 5.8-kb restriction fragment in the wild type following digestion with *EcoRI* (Fig. 2C). These findings were further corroborated by Southern blot (Fig. 2D) using a DNA probe recognizing the region shown in Fig. 2C.

**Figure 3:**
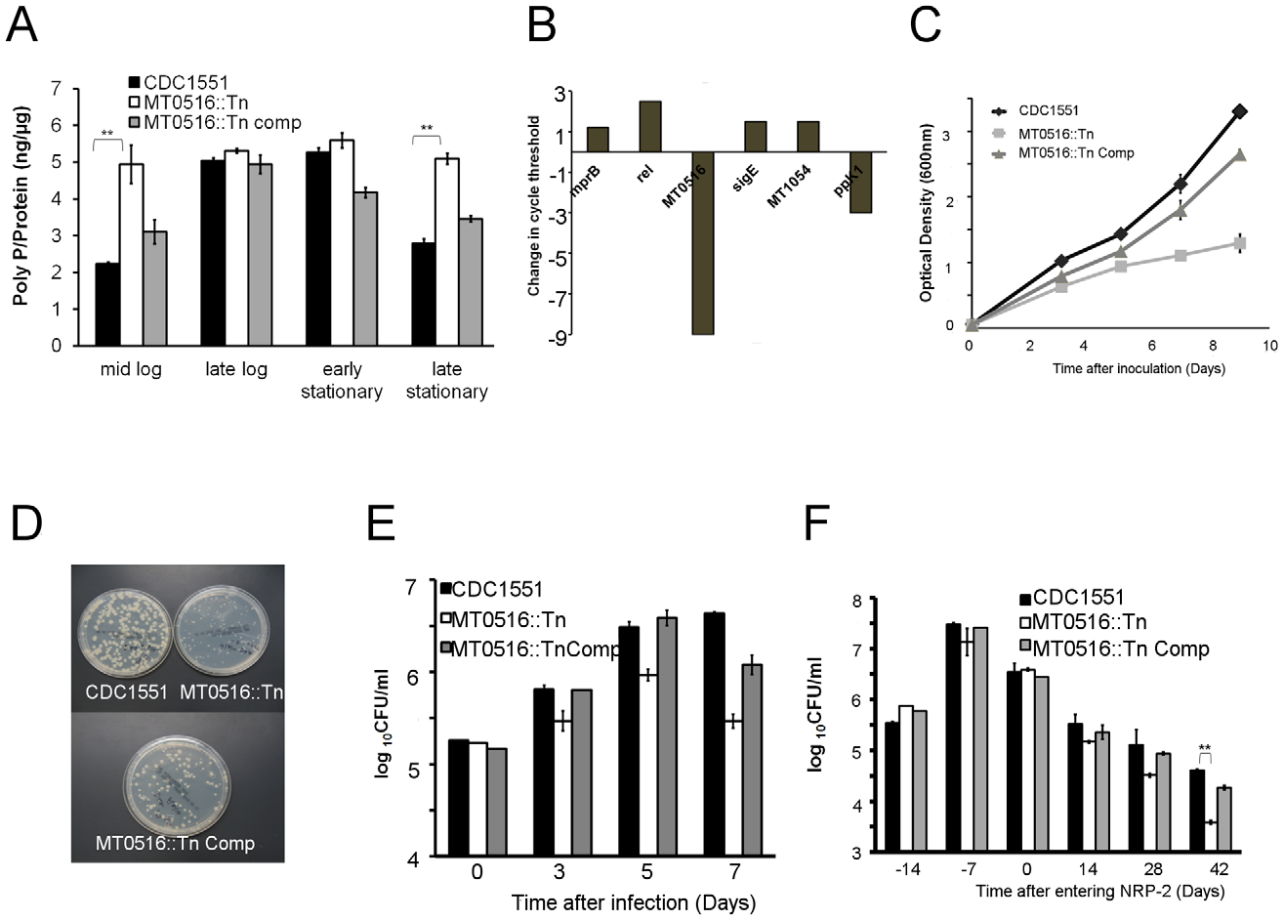
MT0516 deficiency results in persistently elevated intrabacillary levels of poly P and impaired growth in axenic cultures and macrophages. A. Intrabacillary poly P content expressed as ng of poly P per µg of bacterial protein in each strain during different stages of the growth cycle in nutrient-rich broth (**p<0.01). B. RT-PCR analysis of gene expression in MT0516::Tn expressed as change in cycle threshold relative to that of the isogenic wild-type strain during mid-log phase growth in nutrient-rich broth. C. Growth of each strain in nutrient-rich 7H9 broth. D. Colony size of each strain on 7H10 solid media determined 28 days after plating. E. Growth and survival of each strain in THP-1 cells (**p<0.001). F. Growth and survival of each strain during exposure to progressive hypoxia *in vitro* (**p <0.001). CDC1551 =  wild type; MT0516::Tn =  MT0516-deficent mutant; MT0516::Tn Comp =  MT0516::Tn complement strain. CFU  =  colony-forming units. NRP-2 =  Nonreplicating persistence stage 2, as determined by color change of methylene blue dye.

If MT0516 encodes a PPX, we reasoned that deficiency of MT0516 would lead to Mtb poly P accumulation. Mtb poly P was extracted from axenic cultures of MT0516::Tn, MT0516 Comp, and wild-type strains using previously described methods [19], [28], and a DAPI-based fluorescence technique [29] was used to measure intrabacillary poly P. As shown in Fig. 3A, poly P levels were low in the wild-type and MT0516 Comp strains during mid-logarithmic growth phase, reaching a peak during late-logarithmic/early-stationary phase, and then declined again in late stationary phase, as has been described in other organisms [30], [31]. In contrast, the intrabacillary content of poly P was significantly elevated in MT0516::Tn during mid-log phase and was maintained at this level throughout the growth cycle, consistent with the predicted role for MT0516 in poly P hydrolysis.

To determine if increased poly P levels in MT0516::Tn were associated with poly P-mediated signaling, we studied the expression of the *mprAB-sigE-rel* regulatory cascade, which has been shown to be induced in mycobacteria by poly P [19], [20]. Relative to exponentially growing wild-type cultures, growth-phase-matched cultures of MT0516::Tn showed increased expression of *mprB, sigE,* and *rel* by RT-PCR, consistent with poly P-mediated signaling in the mutant (Fig. 3B). Expression of Rv2984/MT3062 (*ppk1*) was downregulated and the expression of Rv1026/MT1054 (putative *ppx*) was upregulated in the MT0516-deficient mutant, consistent with regulatory feedback mechanisms to compensate for elevated intrabacillary poly P levels.

It has been postulated that poly P accumulation may serve as a transient molecular signal for bacterial entry into persistence [13]. We hypothesized that premature poly P-mediated signaling would lead to Mtb growth restriction and phenotypic tolerance to bactericidal drugs. Consistent with our hypothesis, axenic liquid cultures of MT0516::Tn showed decelerated growth relative to that of wild-type and complement cultures (Fig. 3C). Similarly, MT0516::Tn demonstrated a small-colony phenotype on 7H10 solid agar when plates were evaluated 28 days after incubation (Fig. 3D). To determine if MT0516 deficiency is also associated with impaired growth within human monocytes, THP-1 cells were activated and infected with wild type, MT0516::Tn, or MT0516 Comp. Although wild type and MT0516 Comp showed equivalent growth in activated THP-1 cells by Day 5 after infection, MT0516::Tn showed a significant growth defect relative to the wild type and complement strains (p = 0.001), which was further magnified by Day 7 after infection (p<0.001; Fig. 3E).

To determine if poly P accumulation restricts Mtb growth, thereby promoting phenotypic tolerance to antibiotics, we tested the activity of the bactericidal drug isoniazid against MT0516::Tn relative to the wild-type strain. Consistent with the hypothesis that poly P accumulation retards Mtb growth, the MIC of isoniazid against MT0516::Tn increased from 0.03 µg/ml, as observed for the isogenic wild-type strain, to 0.25 to µg/ml (Table 1). On the other hand, MT0516::Tn showed equivalent susceptibility to rifampin relative to the wild-type strain as the MIC remained unchanged (0.25 µg/ml). These studies were performed three times with identical results.

**Table 1:** Sequence of primers used in this study.

Gene/region amplified	Primer sequence
MT0516	Forward5′-cgcggatccggtgcgattggg-3′ Reverse 5′-ccgctcgagtggtttcgtgcc -3′
*kan^R^*	Forward 5′-atgcctcttccgaccatcaagcattt-3′ Reverse 5′-attgcgcctgagcgagacgaaata-3′
*hyg^R^*	Forward 5′-atctcgcggtcgccccgga-3′ Reverse 5′-tgctcaccccccattccgaggt-3′
MT0516 Probe	Forward 5′-ggtgtcgagttgcaggc-3′ Reverse5′-gccaacgagcgaaacgt-3′
*mprB*	Forward-5′-tacccaagcaggagatggtc-3′ Reverse-5′-cataaacctgccacccaatc-3′
*Rel*	Forward-5′-gggtgctggtgataaaggtg-3′ Reverse-5′-aggtcctccaactcccactt-3′
MT0516	Forward5′-cgcggatccggtgcgattggg-3′ Reverse 5′-ccgctcgagtggtttcgtgcc-3′
*sigE*	Forward-5′-accatcacgaccttgagtcc-3′ Reverse-5′-aggtctcctgggtcaggtct- 3′
*ppk1*	Forward-5′-ctcaagacgcactgcaagac -3′ Reverse-5′-tgaacaagtcggtcaagtcg -3′
MT1054	Forward-5′-gactgcggtaccaactcgat-3′ Reverse-5′-tggtgaaacgtcagcagttc-3′

We hypothesized that dysregulated poly P-mediated signaling, in addition to resulting in early growth restriction and antibiotic tolerance, might also impair long-term survival of Mtb under growth-restricting conditions. The wild-type, MT0516::Tn, and MT0516 Comp strains were subjected to progressive hypoxia *in vitro* [32], [33]. By 42 days after entry into nonreplicating persistence stage 2 (NRP-2), when the dissolved oxygen drops to ∼0.06% and bacilli exhibit reduced susceptibility to standard antituberculous drugs [32], [34], MT0516::Tn showed a significant survival defect relative to the isogenic wild-type strain (p = 0.002) (Fig. 3F).

### Poly P hydrolysis is required for M. tuberculosis growth and persistence in guinea pig lungs

**Figure 4:**
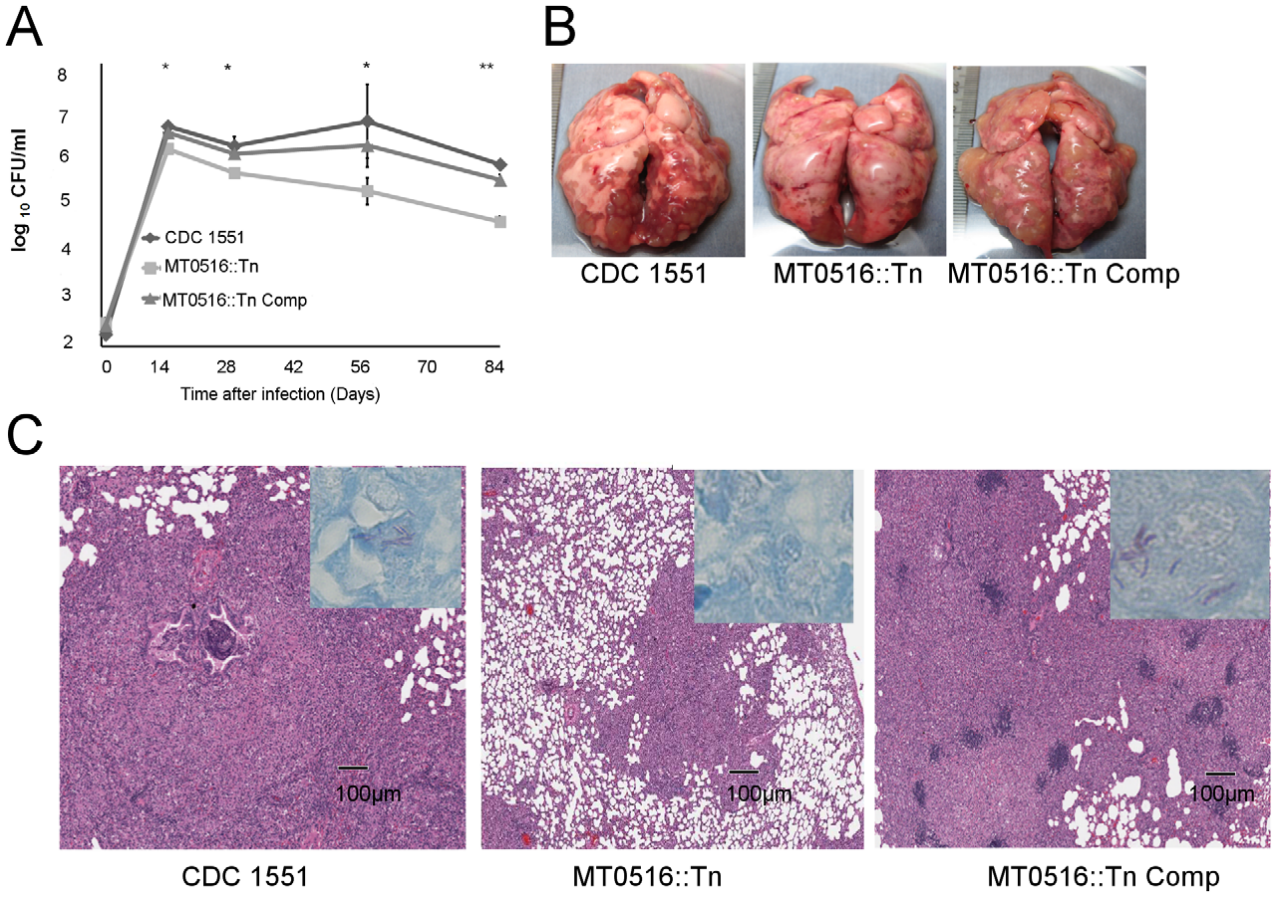
MT0516 is required for full virulence of Mtb in guinea pigs. A. Growth and survival of the mutant following low-dose aerosol infection in guinea pig lungs relative to wild-type and complement strains (*p≤0.01; **p≤0.001 for differences in bacillary counts between mutant- and wild-type-infected lungs). B. Gross pathology of Mtb-infected guinea pig lungs at Day 84 after aerosol infection. C. Microscopic examination of Mtb-infected lungs at Day 84 (H&E stain, 2× magnification. Insets: Ziehl-Neelsen staining, 50x magnification. CDC1551 =  wild-type strain; MT0516::Tn =  MT0516-deficent mutant; MT0516::Tn Comp =  MT0516::Tn complement strain.

The guinea pig model was used to assess the impact of poly P accumulation on Mtb growth and survival within necrotic lung granulomas, which exhibit tissue hypoxia [35], [36]. Guinea pigs were aerosol-infected with wild type, MT0516::Tn, or MT0516 Comp. During the first 14 days after infection, wild-type and MT0516 Comp strains showed typical exponential growth, reaching a peak lung burden of 6.67±0.02 log_10_ and 6.53±0.11 log_10_, respectively (Fig. 4A). On the other hand, MT0516::Tn showed a mild but statistically significant initial growth defect relative to wild type, increasing to only 6.19±0.04 (p = 0.001). After the onset of adaptive immunity, the wild type maintained a relatively stable lung census at Days 56 and 84. The lung CFU of MT0516::Tn-infected animals declined slightly to 5.6±0.07 log_10_ at Day 28 (p = 0.001), decreasing further to 5.29±0.28 log_10_ (p  = 0.01) at Day 56 and 4.64±0.11 log_10_ (p = 0.001) at Day 84. Reintroduction of MT0516 in the complemented strain mostly restored the wild-type growth and survival phenotypes.

All guinea pigs gained weight throughout the experiment, although mean body weight gain between Days 56 and 84 was greatest in the mutant-infected group (454.9±26.8 g to 525.5±39.7 g in MT0516::Tn-infected guinea pigs vs. 442.6±38.9 g to 487.5±55.4 g in wild-type-infected guinea pigs, and 478.8±55.7 g to 483.0±44.3 g in MT0516::Tn Comp-infected guinea pigs). In addition, by week 13 after infection, 3 of 4 animals in the wild-type group, but none in the other groups, became moribund with reduced activity and increased respiratory rate, resulting in early termination of the experiment. Guinea pig lung weights in wild-type and MT0516 Comp groups were 6.99±1.50 g and 7.81±1.75 g, respectively, on Day 56 and 9.92±1.83g and 8.53±0.93 g, respectively, on Day 84. In contrast, lung weights of MT0516::Tn-infected animals were only 4.75±0.81 g on Day 56 and 6.40±0.71 on Day 84 (p = 0.04 at Day 56 and p = 0.01 at Day 84, as compared to wild type). Gross examination of lungs infected with wild-type and MT0516 Comp strains on Day 84 revealed discrete tubercle lesions distributed throughout the lung surface (Fig. 4B), while MT0516::Tn-infected lungs revealed markedly reduced inflammation without discrete lesions.

Histological examination of Day 84 guinea pig samples infected with wild-type and MT0516 Comp strains revealed significant lung involvement, with areas of peribronchiolar inflammation and multiple well-circumscribed granulomas comprising primarily lymphocytes and histiocytes with central necrosis (Fig. 4C). Ziehl-Neelsen staining revealed the presence of multiple acid-fast bacilli in the periphery of granulomas (Fig. 4C inset). In contrast, histological evaluation of MT0516::Tn-infected lung samples revealed significantly reduced inflammation overall (Fig. 4C) and acid-fast bacilli could not be identified in these lung samples.

### Recombinant MT0516 exhibits poly P hydrolysis activity

**Figure 5:**
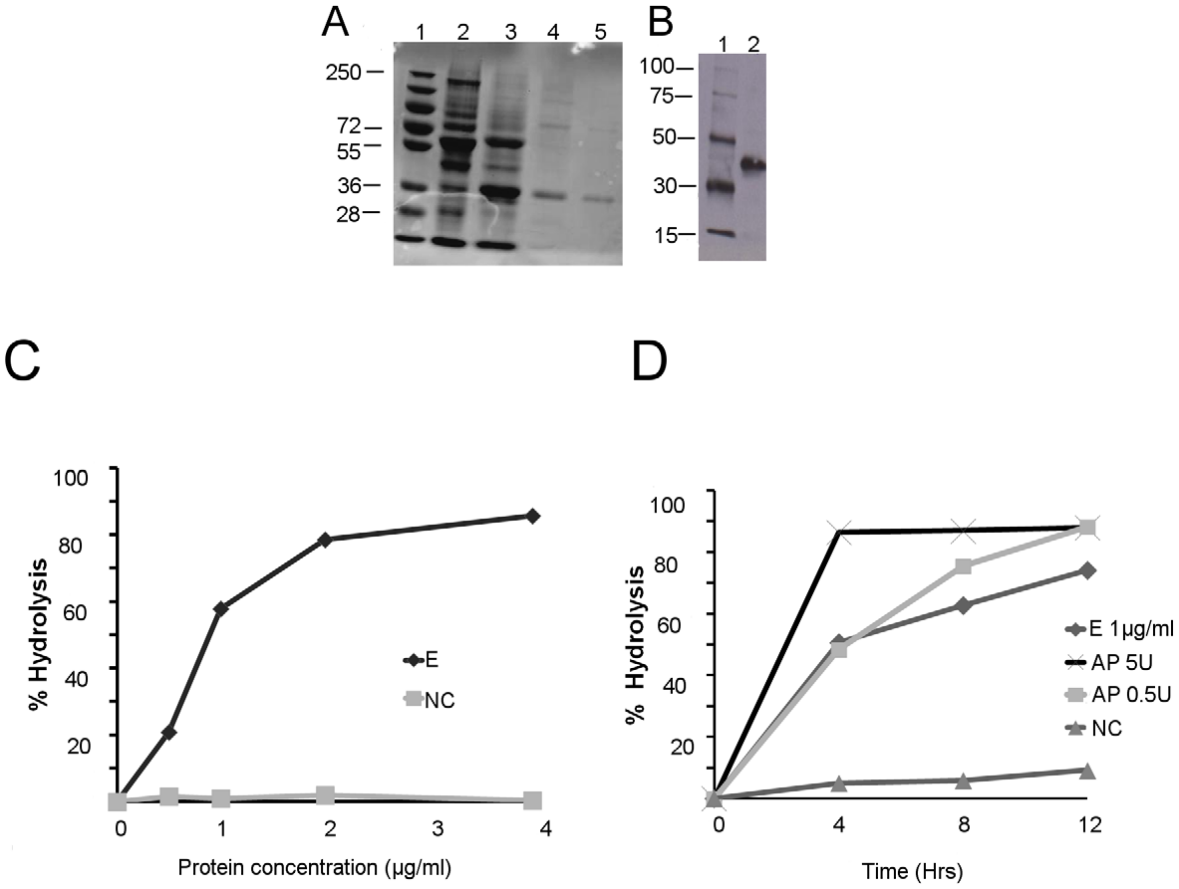
Recombinant MT0516 exhibits concentration- and time-dependent exopolyphosphatase activity. A. Polyacrylamide gel electrophoresis showing the fractions of recombinant His-tagged MT0516 protein (36.6 KDa), expressed in *E.coli* Arctic Express (DE3) BL 21. Lane 1: Molecular weight markers; Lanes 2-3: Supernatant and pellet, respectively, prior to Ni^++^NTA column binding; Lanes 4-5: Elution fraction 1 and 2, respectively, eluted sequentially from Ni^++^NTA column. B. Western blot using Penta-His antibody. Lane 1: Molecular weight markers. Lane 2: Elution fraction 2 after native purification demonstrating the expected size band (36.6 KDa). C. Exopolyphosphatase activity of recombinant MT0516 expressed as % hydrolysis of substrate (65-mer poly P) as a function of protein concentration (μg/ml). **p <0.001. D. Exopolyphosphatase activity of recombinant MT0516 expressed as % hydrolysis of substrate (65-mer poly P) as a function of time (hours). E =  elution fraction 2; AP =  alkaline phosphatase (positive control); NC =  negative control comprising supernatant obtained following IPTG induction of Arctic *E coli* transformed with empty vector. In each reaction, 1 μg/ml poly P was used as substrate. Data for Fig. 5C and 5D are derived from individual experiments, each of which was performed three times with separate samples yielding similar results.

To confirm the activity of MT0516 as a PPX and further characterize its enzymatic properties, we expressed and purified it from *E. coli* as an N-terminal 6X-His fusion protein. A protein of the predicted size (36.6 KDa) was identified in the elution fraction by SDS-PAGE analysis (Fig. 5A), and the identity of recombinant MT0516 was confirmed by Western blot using antibody directed against 6X-His (Fig. 5B), as well as by liquid chromatography tandem mass spectrometry (LCMS; data not shown).

Calf intestine-derived alkaline phosphatase was used as a positive control for the PPX assays. As shown in Figure 5C, recombinant MT0516 demonstrated concentration-dependent PPX activity, as 1 µg of the recombinant protein hydrolyzed ∼57% of poly P (p <0.001relative to negative control), and 2 µg of protein hydrolyzed ∼78% of poly P after 4 hours of incubation with substrate. In addition, the recombinant protein exhibited time-dependent PPX activity (Figure 5D), hydrolyzing ∼51% of poly P by 4 hrs (p<0.001 relative to 0 hrs), and ∼75% of poly P by 12 hrs (p <0.01relative to 4 hrs). On the other hand, an equal concentration of protein obtained from the supernatant of IPTG-induced Arctic *E. coli* transformed with the empty vector revealed no PPX activity (Fig. 5D).

Certain PPXs have a GppA-like activity, cleaving the terminal (γ) phosphate from pppGpp and generating ppGpp [37]. Although modeling experiments did not predict a (p)ppGpp binding cleft in MT0516, we tested the recombinant protein for ppGppase activity. Unlike alkaline phosphatase, recombinant MT0516 did not cleave ppGpp as assessed by HPLC (data not shown). During stress conditions, other bacteria have been shown to increase their poly P content through (p)ppGpp-mediated inhibition of PPX [14]. However, the poly P-hydrolyzing activity of MT0516 was not inhibited by ppGpp (data not shown).

## Discussion

Poly P was originally identified as metachromatically stained “volutin granules” in microbes, and subsequently found to be abundant in every cell in nature [38]. Dismissed as a “molecular fossil” for many years, poly P has been implicated in many important cellular functions, including serving as an alternative source of energy to ATP and reservoir of inorganic phosphate, chelation of cationic metals (e.g., Mn^2+^, Mg^2+^, Ca^2+^), buffer against alkali, environmental remediation of phosphate, and regulation of growth, development, and response to stress conditions [13]. The presence of poly P was documented in Mtb more than five decades ago [39], but its role in Mtb virulence remains uncharacterized. Supporting a potential role for poly P as a growth regulatory molecule in Mtb, recent studies found that that the expression of Rv2984, encoding the poly P kinase PPK1, is induced during stationary phase [19] and inorganic phosphate starvation [40]. In this study, we have identified another mechanism for Mtb regulation of intrabacillary poly P levels through expression of Rv0496/MT0516, which encodes a PPX responsible for poly P hydrolysis.

To our knowledge, this is the first report to establish a direct link between poly P accumulation and phenotypic tolerance of bacteria to antibiotics. This phenomenon was originally described four decades ago [41], and refers to the observation that non-dividing bacteria become less susceptible to killing by cell wall-active antibiotics, which target actively multiplying organisms. In our study, deficiency of the Mtb PPX led to accumulation of poly P and restricted growth in axenic cultures and in human macrophages, as well as reduced susceptibility to killing by the bactericidal drug isoniazid [6], which inhibits the mycolic acid synthesis pathway [7], [8] required for cell wall synthesis. We found evidence of poly P-mediated signaling through the recently described *mprAB-sigE-rel* regulatory pathway [19], suggesting that poly P accumulation triggers the stringent response, which is characterized by dramatically reduced growth, decreased rRNA, tRNA, and protein synthesis, modified RNA polymerase activities, diminished activity of many transport systems, and decreased carbohydrate, amino acid, and phospholipid metabolism [15], [42]. On the other hand, elevated intrabacillary levels of poly P did not alter susceptibility to rifampin, which targets RNA polymerase [43]. The reduced susceptibility of Mtb to isoniazid during human latent TB infection has long been recognized [6], and the relatively greater activity of rifampin against these bacilli is reflected in clinical guidelines, which recommend 9 months of isoniazid or 4 months of rifampin for treatment of latent TB infection [44]. The superior activity of rifampin against nonreplicating bacilli relative to isoniazid also has been observed upon exposure of Mtb to progressive hypoxia [32] and nutrient starvation [45], as well as in animal models of latent TB infection [46], [47]. Whether poly P accumulation alone is sufficient to induce Mtb growth restriction and antibiotic tolerance remains to be proven using an Mtb recombinant strain conditionally overexpressing the *ppk1* gene. The effect of poly P accumulation on bacterial efflux pumps whose induction has been shown recently to induce drug tolerance in mycobacteria [48] is also a subject of future study.

Our data suggest that poly P accumulation may serve as a transient molecular signal for Mtb growth restriction, as intrabacillary levels of poly P peaked in wild-type Mtb prior to entry into stationary phase, as reported in other bacteria [14]. Interestingly, persistently elevated levels of poly P were associated with reduced Mtb persistence during progressive hypoxia and in necrotic granulomas in guinea pigs. Recent studies have shown that antisensing of Rv2984 (*ppk1*) in Mtb leads to reduced intrabacillary poly P content and reduced survival in THP-1 macrophages [19]. Together, these findings suggest that dysregulation of the intrabacillary poly P balance results in reduced Mtb survival during stress conditions. Consistent with tight regulation of poly P levels in bacteria, overexpression of *E. coli ppk* and *ppx* leads to poly P accumulation and depletion, respectively, and, in both cases, to loss of viability [13].

The *E. coli* genes *ppk* and *ppx* comprise an operon, which is regulated by the two-component response regulator PhoB under inorganic phosphate-limited conditions [49]. In contrast, the Mtb genes *ppk1* (Rv2984/MT3062) and *ppx* (Rv0496/MT0516) are located at genetically distinct loci. Expression of *ppk1* in Mtb may be dependent on RegX3, which is a homolog of *E. coli* PhoB and appears to regulate the Mtb phosphate starvation response [40]. On the other hand, despite the genomic proximity of MT0516 to *regX3* (Rv0491/Mt0510), MT0516 expression does not appear to be RegX3-dependent [40]. Bioinformatic analysis suggests that MT0516 is the first gene of a multi-gene operon, which could explain the incomplete restoration of the wild-type phenotype of MT0516::Tn Comp in all assays tested in this study due to potential polar effects on downstream gene expression in MT0516::Tn. Alternatively, native regulation of MT0516 expression may have been altered by integration of MT0516 at the attB site in MT0516 Comp. In other bacteria, accumulation of poly P appears to be mediated through induction of the stringent response and (p)ppGpp-mediated inhibition of PPX activity [14]. In our study, we did not observe inhibition of recombinant MT0516 by ppGpp, suggesting that poly P accumulation in mycobacteria may be regulated primarily through an increase in its biosynthetic rate, as suggested by previous reports [19].

In addition to hydrolyzing poly P, PPX has been implicated directly in bacterial virulence. Recently, a mutant of *Neisseria meningitides* lacking PPX was found to exhibit increased resistance to complement-mediated killing independent of structural changes in the capsule, lipopolysaccharide, and factor H-binding protein, which are known to be required for evasion of the complement system [50]. A *ppx* null mutant of *Bacillus cereus* was found to be defective in motility, biofilm formation, and sporulation efficiency [31]. While most bacteria appear to have a single PPX, deficiency of which leads to a strong mutant phenotype, it seems likely that Mtb has multiple enzymes with PPX activity. Increased expression of MT1054/Rv1026 in the MT0516-deficient mutant raises the interesting possibility that MT1054 can compensate for the lack of MT0516/Rv0496, perhaps limiting the accumulation of poly P and leading to an intermediate phenotype. Current research is focused on characterizing the enzymatic activity of MT1054/Rv1026, and the phenotype of an MT1054 conditional knock-down strain, as well as that of a MT0516/MT1054 double mutant.

In addition to the previously characterized antigens ESAT-6, CFP10, Ag85A, and Ag85B, Rv0496 was identified as a novel secreted T-cell antigen, which is recognized by peripheral blood mononuclear cells derived from close contacts of TB patients with positive tuberculin skin tests [51]. These data suggest that MT0516/Rv0496 is expressed by *M. tuberculosis* during human latent TB infection, raising the possibility that this antigen, like ESAT-6 and CFP10, could be used to diagnose latently-infected individuals. Although poly P is present in all cells, enzymes homologous to Mtb PPX are absent in eukaryotes, suggesting that the latter may represent an attractive drug target. Since PPX is required for Mtb persistence in necrotic lung lesions, it is possible that a small molecule inhibitor to this enzyme may target “persister” bacilli, thereby contributing to shortening of TB treatment.

## Materials and Methods

### Ethics Statement

All procedures involving animals were performed according to protocols approved by the Institutional Animal Care and Use Committee at the Johns Hopkins University (JHU protocol approval number GP09M68). All guinea pigs were maintained and bred under specific-pathogen-free conditions and fed water and chow ad libitum.

### Bacterial strains and growth conditions

The JHU standard reference strain of *M. tuberculosis* CDC 1551 strain was used in all experiments as the wild-type control and was the host strain used for mutagenesis with the *Himar1* transposon, as previously described [27]. A mutant with a transposon insertion in MT0516/Rv0496 (MT0516::Tn) was identified in our archived library, and the transposon insertion site was determined to be at bp 732 (total gene length  =  1035 bp), as previously described [27], [52]. For construction of the complement strain (MT0516 Comp), genomic DNA was isolated from *M. tuberculosis* CDC1551 according to standard protocols [53]. A 1235-bp region encompassing the MT0516 gene and 200-bp sequence upstream of the putative transcription start sequence was PCR-amplified using forward primer 5′-GGG GAC AAG TTT GTA CAA AAA AGC AGG CTT CCC ACC CAC TCA CGG GCG AA-3′and reverse primer5′- GGG GAC CAC TTT GTA CAA GAA AGC TGG GTC CTG GCC AGC AAC TCG GC- 3′. The amplified sequence was cloned into pDONR vector (Invitrogen) and then into the Entry vector pGS202 by a series of homologous recombination steps using the Gateway cloning system (Invitrogen) and transformed into *E. coli* DH5α according to the manufacturer's instructions. Transformants were selected on hygromycin-supplemented LB agar and the identity of the clones was confirmed by DNA sequencing. Plasmid DNA from positive clones was electoporated into MT0516::Tn competent cells according to standard protocols, as previously described [53]. The transformants were plated on Middlebrook 7H10 plates supplemented with hygromycin (100 µg/ml) and kanamycin (50 µg/ml). The identity of MT0516 Comp was confirmed by PCR and Southern blot (Fig. 2).

Each strain was grown in Middlebrook 7H9 broth (Difco, Sparks, MD) supplemented with 10% oleic acid-albumin-dextrose-catalase (OADC, Difco), glycerol, 0.05% Tween 80 at 37°C on a roller. The growth of each strain was assessed at designated intervals by measuring the optical density at 600nm and by plating samples on Middlebrook 7H10 plates (Difco, Sparks, MD). Colony-forming units (CFU) were enumerated after incubation of plates for 4 weeks at 37°C, and the colony morphology was recorded.

### Cloning, expression, and purification of recombinant His-tagged MT0516

A 1035-bp region encompassing the full-length MT0516 gene was amplified from *M. tuberculosis* CDC1551 DNA using forward primer 5′-ccgctcgaggtgcgattgggcgtgct -3′ and reverse primer 5′- cgcggatcctcatggtttgctgcctc -3′and the Xho I-BamH I-digested fragment was cloned into T7 plasmid pET15b containing an N-terminal 6X-His tag (Novagen). The resulting expression plasmid, MT0516/pET was used to transform *E. coli* Arctic Express (DE3) RP competent cells (Stratagene). Empty vector lacking the MT0516 gene were used to mock transform and construct negative control strain. The transformed bacteria were plated and colonies were isolated on LB agar supplemented with ampicillin (100 µg/ml). Positive clones were confirmed by sequencing and were grown in LB broth supplemented with gentamycin (20 µg/ml) and ampicillin (100 µg/ml) and the recombinant protein was overexpressed upon induction with 1mM IPTG. Cells were harvested after 24 hours of induction at 9°C and stored overnight at -20°C. Mock-transformed cells were also induced with 1mM IPTG and the cell supernatant was dialyzed and used as negative control in subsequent experiments.

The protein was purified under native conditions according to standard protocol (Qiagen). The dialyzed active protein fractions were quantified using Bradford reagent (Sigma) and analyzed by SDS-PAGE. A Western blot was performed to confirm the identity of recombinant MT0516 using Penta His Antibody (Qiagen) directed against 6X-His according to the manufacturer's instructions. The active fractions were frozen and stored at −80°C until use.

### Analysis of recombinant MT0516 by mass spectrometry

The 36.6 KDa band from SDS gel and the elution fraction after native purification of recombinant protein were reduced and alkylated with iodacetamide and proteolyzed with sequencing-grade modified procine trypsin (Promega). Protein identification by liquid chromatography tandem mass spectrometry (LCMS) analysis of peptides was performed using an LTQ ion trap MS (Thermo Fisher scientific), interfaced with a 2D nanoLC system (Eksigent). Peptides were fractionated by reverse phase HPLC on a 75 µm X100mm C18 column with a 10 µm emitter using 0-60% acetonitrile/0.1% formic acid gradient over 90 min at 300nl/min. Peptide sequences were identified using Mascot software to search the NCBI nr 167 database with acquired raw MS data, with oxidation on methionine and carbamidomethylation on cysteine as variable modifications. Mascot search result *.dat files were processed in Scaffold to validate protein and peptide identifications.

### Extraction and measurement of intrabacillary poly P content

Mtb strains MT0516::Tn, MT0516 Comp, and the isogenic CDC1551 wild-type strain were grown in Middlebrook 7H9 broth (Difco, Sparks, MD) supplemented with 10% oleic acid-albumin-dextrose-catalase (OADC, Difco), glycerol, 0.05% Tween-80,at 37°C on a roller. At regular intervals samples were collected and poly P was extracted. Briefly, bacterial cultures were diluted to an OD of 0.45to 0.5 at regular time points, pelleted and lysed using 5M guanidine isothiocyanate (GITC)-50 mM Tris-HCl, pH 7.0 (GITC lysis buffer) prewarmed to 95°C. Following brief sonication, samples from each experimental group and control were removed for protein estimation [54]. Poly P was purified using glass milk beads from the Geneclean III kit (MP Biomedicals LLC) and eluted in warm water (pH 8.0) [13], and the eluted mixture was treated with DNase and RNase. PCR of the *sigA* gene confirmed the absence of detectable DNA contamination in all samples.

A DAPI (4′,6-diamidino-2-phenylindole)-based assay capable of detecting poly P concentrations in the ng/ml range [29] was used for poly P measurements. Briefly, the flourescent dye DAPI binds to poly P and when the DAPI-polyP complex is excited at 415 nm, peak fluorescence is emitted at 525 nm. At this wavelength there is very little background emission from either free DAPI or DAPI-DNA complex [29]. The minimum concentration of poly P detected by this method is 25 ng/ml. For the generation of a standardized curve, 1 µg/ml DAPI (Roche Diagnostics) was incubated for 10 min at room temperature with increasing concentrations of poly P (average 65-mer, Sigma). The shift in fluorescence was measured by FLUOstar OPTIMA (BMG LABTECH) at 415nm (excitation) and 525nm (emission) wavelengths.

### Assay of poly P and (p)ppGpp hydrolysis activity of recombinant MT0516

The pooled, dialyzed elution fraction containing recombinant MT0516 was used to assay for poly P hydrolysis activity. Briefly, the elution fraction containing 1.0 µg/ml of recombinant MT0516 was incubated with 1 µg/ml of 65-mer poly P in 50 mM Tris HCl buffer (10 mM MgCl_2_,1.5 mM KCl) pH 8.0 for 12 hr. Calf intestine-derived alkaline phosphatase (100 U, New England Biolabs) was used as a positive control for these reactions. The poly P concentration of triplicate samples was quantified at designated time points, using the DAPI-based fluorescence assay described above.

To determine the effect of ppGpp on the activity of recombinant MT0516, the reaction was repeated with addition of 10 µM ppGpp and incubated for 12 hrs before measuring residual poly P in the mixture. ppGpp (1 µM; TriLink BioTechnologies, San Diego, CA) was incubated with the elution fraction of recombinant MT0516 (1 µg/ml) for 30 min in 50 mM Tris HCl buffer pH 7.5. Antarctic phosphatase (100 U) was used as a positive control. 20 µl of sample from each reaction was injected into a reverse-phase HPLC (Waters 2690) fitted with Sunfire C18 column and a UV detector (Waters 2487), to study the hydrolysis of ppGpp and the resulting peaks were analyzed with Chrom Perfect software.

### Gene expression analysis by RT-PCR

50-ml samples were removed from logarithmically-growing cultures at mid-log phase, (OD = 0.45-0.5) and centrifuged at 3500 rpm for 10 minutes at 4°C. The supernatants were removed and the pellets were resuspended in 1 ml TriZOL reagent (GIBCO/BRL). Mycobacterial membranes were disrupted using 0.1 mm zirconia/silica beads in a Bio-Spec bead beater (8 cycles x 30 sec/cycle). Mycobacterial RNA was recovered by centrifugation, chloroform extraction, and isopropyl alcohol precipitation, as previously described [46]. Prior to reverse transcription, control and mutant RNA (10 ng) were treated with RNase-free DNase (Invitrogen) and subjected to 36 cycles of PCR to ensure that all DNA had been removed, as assessed by ethidium bromide-stained agarose gel analysis. Fluorescently-labeled cDNA was generated using Superscript III (Invitrogen), using fluorescent dyes Cy3 and Cy5 (Amersham). cDNA corresponding to each transcript was subjected to 34 cycles of PCR for quantification using the primers listed in Table 1. The cycle threshold value (C_T_) obtained for each gene of interest (GOI) was normalized with that of *sigA*, a housekeeping gene (HKG) with constant expression under different experimental conditions [55], in order to obtain the normalized C_T_ (nC_T_  =  (GOI C_T_) – (HKG C_T_)). The change in C_T_ (ΔC_T_) was calculated using the following formula: (ΔC_T_  =  C(nC_T_) – S(nC_T_)), where C represents the control strain and S represents MT0516::Tn. The experiment was repeated three times with similar results and the figure is representative of one experiment.

### Progressive hypoxia model of infection

Each strain was diluted to ∼10^4^ bacilli/ml, as determined by OD_600_. Cultures were inoculated into individual test tubes containing a magnetic stir bar ratio and a head-space air to medium volume (HSR) of 0.5 and sealed with rubber stoppers. The cultures were incubated upright at 37°C with stir-bars spun at a speed sufficient for uniform suspension of organisms without agitating the surface of the medium. The reduction and decolorization of the methylene blue dye served as a visual indicator of oxygen depletion sufficient to induce bacillary entry into NRP stage 2 [32]. At designated time points, samples were collected by piercing the rubber stoppers without introducing atmospheric oxygen into the tubes, as assessed by maintenance of the decolorized dye state.

### Antibiotic exposure

Wild-type and MT0516::Tn strains were grown to mid-exponential phase in supplemented Middlebrook 7H9 broth. For determination of isoniazid minimum inhibitory concentration (MIC), 10^5^ organisms from each culture were grown for 14 days at 37°C in the presence of the following final concentrations of isoniazid: 0, 0.03, 0.06, 0.12, 0.25, 0.5, 1, 2, 4, and 8 µg/ml. For determination of rifampin MIC, 10^5^ organisms from each culture were grown for 14 days at 37°C in the presence of the following final concentrations of rifampin: 0, 0.125, 0.25, 0.5, 1, 2, 4, and 8 µg/ml. Samples were incubated in standing tubes and the MIC was defined as the lowest antibiotic concentration in which a bacterial pellet was absent.

### Growth kinetics in human macrophages

The growth of each strain in activated THP-1 cells was assessed as previously described [56] with slight modifications. Each strain was grown to mid-logarithmic phase in 7H9 Middlebrook broth and diluted 100-fold, pelleted, washed twice in RPMI (GIBCO) supplemented with 10% FBS and resuspended in complete RPMI without antibiotics. Phorbol 12-myrisate-13-acetate (20nM)-activated THP-1 cells (ATCC) were infected with individual Mtb cultures for 2 hours. The cells were washed twice with fresh RPMI supplemented with 10% FBS and incubated with fresh antibiotic free-media. The cells were lysed with Triton X-100 at predetermined time points and the lysate was plated on Middlebrook 7H10 plates for colony enumeration of intracellular bacteria.

### Animal studies

Female outbred Hartley guinea pigs (250–300 g) were purchased from Charles River Labs (Wilmington, MA). Separate groups of 20 female Hartley guinea pigs were infected with a Madison chamber aerosol generation device (College of Engineering Shops, University of Wisconsin, Madison, WI) calibrated to deliver approximately 100 bacilli of MT0516::Tn, MT0516 Comp, or wild type into guinea pig lungs, as previously described [57]. Four guinea pigs from each group were euthanized at Days 1, 14, 28, 56, and 84 after infection. At necropsy, lungs and spleens were removed aseptically, weighed, and examined. Organs were then homogenized for CFU enumeration and processed for histology. Lungs were homogenized in 10–15 ml PBS using a Kinematica Polytron Homogenizer with a 12-mm generator (Brinkman) within a BSL-III Glovebox Cabinet (Germfree), as previously described [33], [58]. Serial tenfold dilutions of organ homogenates were plated on 7H11 selective agar (BBL) except for Day 1 lung samples, which were plated neat. Plates were incubated at 37°C and CFU were counted 4 weeks later. At various time points after infection, lung samples were fixed in 10% PBS-buffered formalin, paraffin-embedded, and sectioned. Sections were stained with hematoxylin/eosin and Ziehl–Neelsen for microscopy. The histopathological sections were reviewed by two independent observers who were blinded to the study groups.

### Statistical analysis

The mean of log-transformed lung CFU values, based on four samples for each measure, were compared by the Student *t*-test. A *p*-value of < .05 was considered statistically significant. The error bars represent the Standard Deviation (SD). Statistical analysis of gene expression studies was performed using three biological replicates of each sample at each time point. Synthesized cDNA was subjected to three technical replicates of PCR amplification for each biological sample. Each individual reaction was compared to a reaction lacking reverse transcriptase (background signal). Melt curves were generated for each reaction and successful reactions contained only one melt peak above threshold, representing a single amplicon per reaction. Normalized technical replicate values were averaged to generate a single value for each biological sample. Mean and standard deviation (SD) were then calculated for the three biological replicate values of each gene of interest.
